# PHARMIP: An insilico method to predict genetics that underpin adverse drug reactions

**DOI:** 10.1016/j.mex.2019.100775

**Published:** 2019-12-19

**Authors:** Ahmad M. Zidan, Eman A. Saad, Nasser E. Ibrahim, Amal Mahmoud, Medhat H. Hashem, Alaa A. Hemeida

**Affiliations:** a Department of Bioinformatics, Genetic Engineering & Biotechnology Research Institute, University of Sadat City, Egypt; b Department of Biology, College of Science, Imam Abdulrahman Bin Faisal University, P.O. Box 1982, 31441, Dammam, Saudi Arabia; c Department of Animal Biotechnology, Genetic Engineering & Biotechnology Research Institute, University of Sadat City, Egypt

**Keywords:** Pharmacovigilance/ pharmacogenomics insilico pipeline (PHARMIP), Pharmacovigilance, Pharmacogenomics, Precision medicine, Similarity ensemble approach, Pharmacophore mapping, Molecular docking, Bioinformatics

## Abstract

Pharmacovigilance is the pharmacological science that focuses on the safe and appropriate use of drugs.Variability in response to drug therapy in both terms of safety and efficacy is highly related to patient's personal genomics. Hence, pharmacovigilance considers pharmacogenomics methodologies in the evaluation of medicinal products. The aim of this work is to introduce the pharmacovigilance/ pharmacogenomics insilico pipeline (PHARMIP) that uses the drug (or drug candidate) digital structure and the advances in bioinformatics tools and databases to figure-out the genetic factors underlying the drug reported adverse reactions (ADRs).PHARMIP uses user-friendly freely available bioinformatics resources to help pharmacovigilance and pharmacogenomics scientists with minimal bioinformatics experience to retrieve helpful information for their daily basis activities. Also, PHARMIP could help the advances in precision medicine in a drug-centric approach as it can be used to reveal genetic risk factors for certain drug ADRs. Domperidone was used as an example to the application of PHARMIP as the pipeline was initially developed during the insilico exploration of domperidone cardiotoxic ADRs.

Method is composed of 3 main steps:

• Preparing the drug off-label targets (OLT) list.
• Retrieving the related diseases/ adverse reactions (DA) list.
• Analysis of DA list to get answers.

## Graphical abstract

**Figure:**
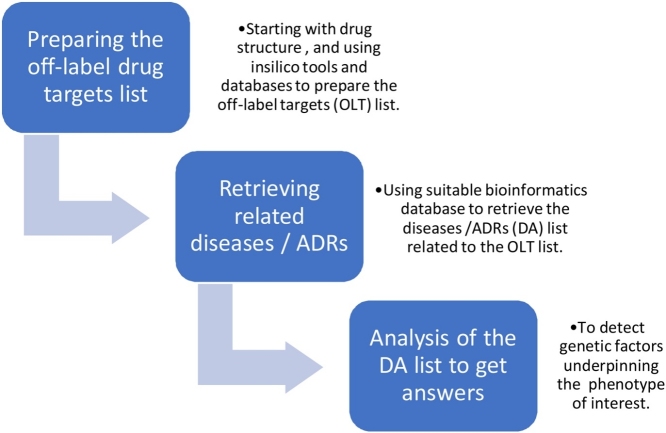

**Specification Table**

**Table:** 

Subject Area:	Pharmacology, Toxicology and Pharmaceutical Science
More specific subject area:	In-silico pharmacology
Method name:	Pharmacovigilance/ pharmacogenomics insilico pipeline (PHARMIP)
Name and reference of original method:	If applicable, include full bibliographic details of the main reference(s) describing the original method from which the new method was derived.
Resource availability:	• Binding DB link • CTD analyzer link • DisGeNET link • DrugBank link • HGNC link • KEGG disease mapper tool link • OMIM link • OpenBabel converter link • PDB link • PharMapper link • Polypharmacology browser link • Pubchem sketcher link • PyRX download link • SEA server link • STRING DB link • SwissTargtPrediction link • UniProt retrieve ID tool link • VigiAccess link • ZINC database link

## PHARMIP at a glance

The method is composed mainly of three pipelined steps starting with the chemical structure of the drug or the drug candidate with the aid of free user-friendly bioinformatics tools and databases to get a list of candidate genes and genetic variants that may underpin an adverse reaction. We developed this method to address the recent requirements in pharmacovigilance and pharmacogenetics. To the best of our knowledge, there is no free software or bioinformatics tool that do the same job. And, we hope that this pipeline could be programmed into a tool in a future work. The predicted time to run the PHARMIP pipeline is variable and mainly depends on the step of PharMapper as a rate-limiting step. PharMapper job could range from several days to several weeks as the server runs the jobs in a queue. Limitations of PHARMIP could include the instability of the bioinformatics databases as every update of the database will change the results leading to some problems of research reproducibility. Also, some docking aspects and basics could be a complex issue for the audience of this work (pharmacovigilance and pharmacogenomics scientists). So, we tried to use the simplest free software with the default option to avoid technical details that could be inappropriate for the bioinformatics-naïve audience. Each tool or database included in this work has its own manual and/or FAQ to troubleshoot the common problems. We encourage the readers to refer to these manuals in case they faced any problems using these tools.

## Method details

### Predicting the off-label drug targets

#### Similarity ensemble approach (SEA)

Starting with the chemical structure of the drug, the off-label targets could be predicted by the similarity ensemble approach (SEA). This approach predicts new protein targets based on chemical similarity between ligands [1]. Most tools that use this approach queried bydrug or candidate structure in the Simplified Molecular-Input Line-Entry System (SMILES) format [2]. There are many chemical databases to retrieve such information. However, for drugs the easiest way is to retrieve the SMILES code from the drug page at the DrugBank database [3]. If the ligand is a new structure as in case of a drug candidate or a new molecule, the SMILES code could be retrieved using molecular drawer like Pubchem sketcher or other suitable tool that allow deriving the SMILES code from directly drawing the chemical structure. Also, any other chemical format for the input molecule could be easily converted to SMILES using chemical format converter like OpenBabel converter. Once the ligand SMILES is ready, it could be used to feed several tools that use the SEA.

Several tools use SEA to assign new targets to the query molecule based on its chemical similarity score to other chemicals (usually referred to as Tanimoto coefficient or similarity score). Each tool screens its target chemical repository using different SEA models to retrieve hits. Biological targets of these hits are assigned to the query ligand depending on SEA. Targets from different tools could then collected and filtered using suitable significant level (e.g. P-value of 0.01 or 0.05).

Some publicly available and user-friendly SEA tools are listed below:

• SEA server [1].
• SwissTargetPrediction server [4].
• Polypharmacology browser (PPB) [5].

One advantage of Polypharmacology browser is that it retrieves the already reported query molecule bioactivity in ChEMBL database [6]. Then, it predicts targets based on 6 different fingerprints and 4 combination of fingerprints. Using PPB enriches the off-label targets list with targets that have wet-lab evidences.

#### Reverse pharmacophore mapping

Pharmacophore of a drug is the set of molecular features that cause it to be identified by its biological target [7]. The normal pharmacophore mapping retrieves ligands whose pharmacophore fit certain protein target. The reverse pharmacophore retrieves protein targets for a certain ligand. A very famous and freely available insilico tool that uses reverse pharmacophore mapping approach is PharMapper server [8]. PharMapper needs the query molecule in MOL2, or SDF chemical file formats. While SDF format is available in DugBank, MOL2 could be retrieved from ZINC database [9]. There are different options to customize the tool before job submission. Default options resemble the optimum choice for non-specialists. The job may take one to two weeks to spit-out results according to jobs queue length. So, the option of getting job results alert by email is worthy.

**Fig. 1:**
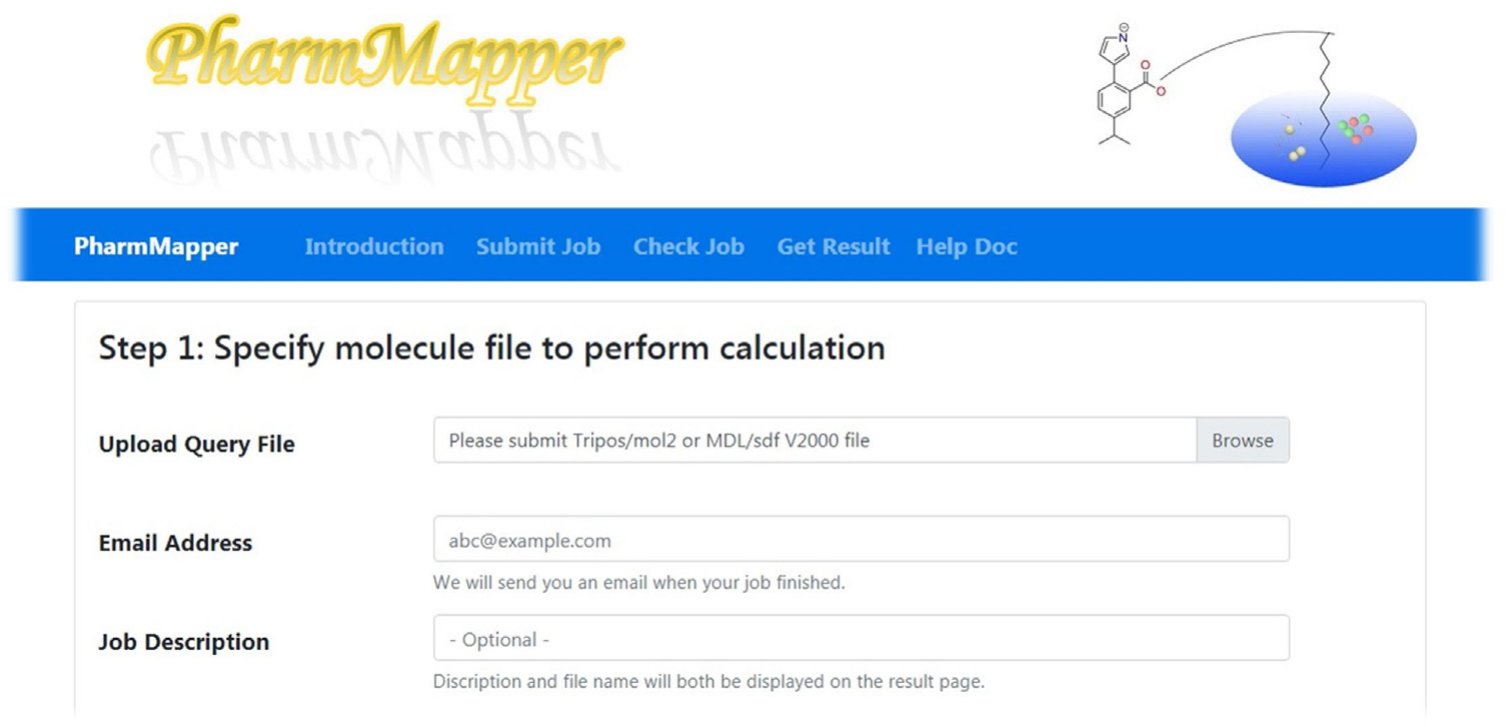
The user interface of PharMapper tool.

The results will retrieve 300 targets by default. These targets could be arranged using different parameters. Z -score of the results could be used to detect which of the 300 results will be picked up to the targets list based on the significance level of choice (e.g. for P-value of 0.01 select targets withZ-score ≥ 2.326, and for P-value of 0.05 select targets with Z-score ≥1.645) (Fig. 1).

#### Molecular docking

Docking is a featured insilico technique that is usually used to predict the best orientation of a ligand when bound to the active site of a protein [10]. Pharmacophore mapping usually outperforms docking in virtual screening to predict new ligand-target relationships [11]. However, docking improves and evaluates predictions of pharmacophore [12] and SEA [13]. The main use of molecular docking in this pipeline is to evaluate the affinity of the drug to the predicted targets by SEA and pharmacophore mapping. One of the featured tools for molecular docking is Auto Dock Vina [14] which can be used as a standalone program or imbedded in a platform like PyRX [15].

Docking jobs using PyRX needs the drug in the SDF format and the target protein in 3D structured format. Normally, the 3D structure of a protein could be retrieved from the PDB database [16]. Some targets still have no experimentally determined 3D structure in PDB database. In this case, homology modeled 3D structure for these targets could be then obtained from SWISS-MODEL database [17]. From the Vina wizard in PyRX, the drug SDF file is added as a ligand and the protein target 3D structure file is added as a macromolecule.

Sometimes, PyRX doesn't accept a certain 3D protein structure for docking due to some technical errors. Each protein has different entries and identifiers in 3D protein structure databases. All these entries could be found in the protein's Uniprot page in the cross-references and/or structure sections.

The results of this step will be the best conformation preference of the ligand into the binding site of the target protein. AutoDock Vina normally predicts the free binding energy (also known as Gibbs free energy or ΔG) in kcal/mol. For calculating the affinity of the drug to the predicted target, the dissociation constant K_d_ could be calculated from the free binding energy using the python script from [18]. In PyRX python shell, the code is run, free binding energy from docking is entered in kcal/mol, and the K_d_ is calculated in moles. Binding DB [19] is a good source to compare the drug predicted affinity in terms of K_d_ to the experimental affinities obtained in wet lab settings.

The end result of this pipeline step will be a list of off-label targets (OLT list) obtained by SEA, pharmacophore mapping then validated using molecular docking. Target list identifiers may be in different formats according to the tool used. The combined list of targets obtained from different tools could be refined and unified by the retrieve/ID mapping tool from the UniProt database [20]. Visualization and different analyses could be done on the list using STRING database [21]. The final OLT list will be used as an input for the next step to retrieve the related diseases/ phenotypes list.

### Retrieving the diseases /ADRs (DA) list related to OLT list

Using the OLT list from the previous step, different databases could be used to retrieve diseases and / or ADRs linked to these targets. Online Mendelian inheritance in man (OMIM) database [22], disease-gene network (DisGeNET) database [23], and comparative toxicogenomic database (CTD) [24] are good examples of these databases. Also, Kyoto encyclopedia of genes and genomes (KEGG) [25] has a disease mapper tool that could be fed by a list of gene identifiers to retrieve a list of related diseases.

The first overview of the OLT list could be done by the enrichment analysis. CTD offers the option of disease enrichment analysis which could be done to figure out the types of diseases or side effects that could result from the drug or candidate use. A certain disease is considered statistically enriched if the proportion of its related genes to the full enquiry gene list is larger than the proportion of all its related genes to the whole genome [26]. This type of analysis could be done on the level of biochemical pathways and / or gene ontology functional terms. This is very helpful in foreseeing the complete predicted picture of the query drug and its biological effects on several levels.

DisGeNET has the option of retrieving associations at the genetic variant level. Normally, DisGeNET retrieves results at gene level ranked by gene-disease association (GDA) score, and at genetic variant level ranked by variant-disease association (VDA) score. Retrieving genetic variants linked to certain drug-disease association could resemble a draft for drug's pharmacogenetics variants network that could be used in several ways. For example, detecting biomarkers related to a certain ADR, genetic guided patient recruiting in drug clinical trials, and drug genetic labelling experiments.

**Fig. 2:**
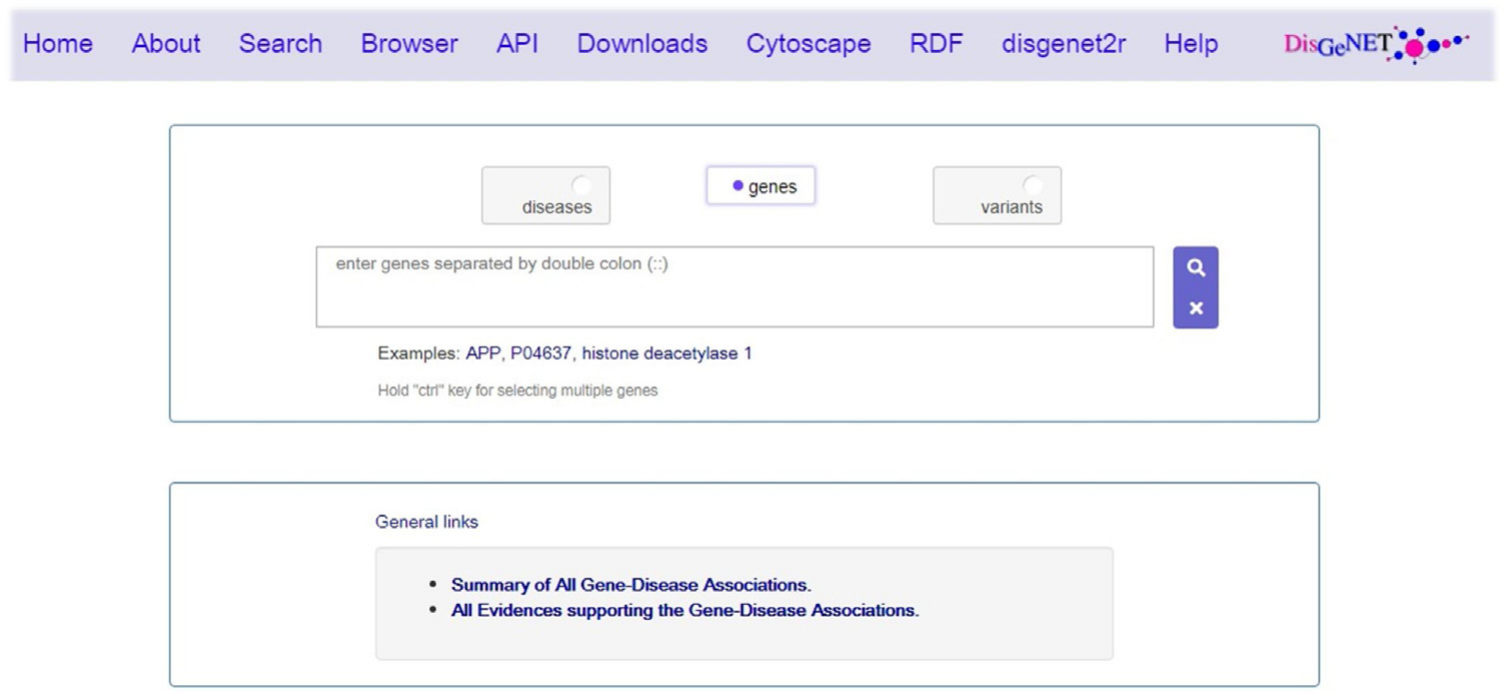
The user interface of DisGeNet database.

The results of this step will be a list of diseases and ADRs (DA list) related to the OLT list (Fig. 2).

### Analysis of DA list to get answers

DA list from the previous step could be analyzed in several ways according to the question to be answered. Normally, these results could be used either to predict or to explain the genetics underpinning for a certain side effect of the enquired drug.

In Pharmacovigilance, healthcare providing entities usually report ADRs noticed during using a certain drug. These individual case safety reports (ICSRs) are usually saved in specialized databases to be analyzed. One of the featured ICSRs databases is the world health organization (WHO) database VigiBase [27] which is freely accessed through the VigiAccess portal. Analyses of pharmacovigilance data include signal detection and different risk management activities. Recently. European medicines agency (EMA) released a guideline on the use of pharmacogenomics methodologies in the pharmacovigilance regular activities. The guideline describes 3 types of genomic biomarkers (BM) that influence drug safety and efficacy. Namely, they are pharmacokinetic (PK) BM, pharmacodynamic (PD) BM, and BM associated with drug-induced toxicity risk [28]. PHARMIP uses the power of insilico tools and bioinformatics databases to figure out the different genomics BM underlying drug safety and efficacy issues.

In pharmacogenomics, regular activities include discovery, evaluation, and implementation of genetic BM influencing drug response. These data are saved in specialized databases that provide information about gene-drug associations (e.g. PharmGKB [29]). PHARMIP could be used to assist pharmacogenomics daily activities via detection of candidate genes and variants that influence certain drug response.

In precision medicine, pharmacogenomics is used as a tool for genetic guided therapy personalization. PHARMIP, via its role in pharmacogenomics, could help advances in precision medicine from a drug-centric point of view. Construction of drug-centered gene network could reveal insights about the precise use of this drug with different patients' genetic profiles.

The following are two examples of using PHARMIP to detect the underpinning genetics of some reported domperidone cardiotoxic ADRs and ranitidine related thrombocytopenia.

### Example 1: domperidone related cardiotoxic effects

Domperidone (DrugBank accession no. DB01184) is a specific dopaminergic blocker approved for treatment of several gastrointestinal disorders like nausea, vomiting and emesis. It's used to treat gastroparesis by its D2 selective antagonism that causes increase in GIT peristalsis. It's also used as a galactagogue by increasing prolactin secretion as a part of its anti-dopaminergic effect [30,31]. It is also used to relieve the gastrointestinal symptoms of Parkinson's disease and in some cases as an unintended antipsychotic drug [32,33].

Although being successfully used for about 40 years as a prokinetic drug in different gastrointestinal motility disorders, domperidone was repeatedly reported to have cardiotoxic effects. Leelakanok et al. [34] reported a study results that domperidone increases the risk of cardiac arrhythmia and sudden cardiac death by 70 %. Similar results were also reported by Johannes et al. [35] and van Noord et al. [36]. This cardiac toxicity is mainly thought to be resulting from the drug effect on cardiac QT interval which can increase the risk of Torsades de Pointes [37]. Also, domperidone dependent ventricular arrhythmias were reported. It is thought to be an effect of blockade of hERG voltage-gated potassium channels [38,39].

Domperidone VigiAccess page contains 9201 ICSRs in 27 categories (on 21^st^ January 2019). Among these ICSRs, 584 reports are for cardiac disorders in 50 subcategories. Some of these cardiac disorders are heavily reported (e.g. palpitation has 171 ICSRs) and others are seldomly reported (e.g. atrial flutter has only 1 ICSR).

Cardiotoxic ADRs that will be investigated by PHARMIP in this example are:

• Arrhythmias (88 ICSRs in VigiBase)
• Cardiac arrest (48 ICSRs in VigiBase)
• Torsades de Pointes (32 ICSRs in VigiBase)
• Long QT syndrome (5 ICSRs in VigiBase)

### Predicting the off-label drug targets (OLT list)

#### SEA

Domperidone SMILES code (ClC1=CC2=C(C = C1)N(C1CCN(CCCN3C(=O)NC4=CC = CC = C34)CC1)C(=O)N2) was retrieved from its DrugBank page then fed to SEA tools. Different targets were predicted using this approach. All targets are presented in human genome ontology gene nomenclature committee (HGNC) symbols [40].

• SEA server searches hits in the largest chemical dataset (ZINC15) and retrieved 87 targets with significant P-values. Some of these targets were non-human. After removing redundancy and non-human targets, 51 targets remained:

(ACLY,ADRA1A,ADRA1B,ADRA1D, ADRA2A, ADRA2B, ADRA2C, AKT1, AKT2, AKT3, CACNA1G, CALCRL, CHRM1, CHRM2, CHRM3, CHRM4, CHRM5, CTSS, CYP2D6, DRD2, DRD3, DRD4, GLRA1, GRIN1, GRIN2B, HRH1, HTR1A, HTR2A, HTR2B, HTR2C, HTR3A, HTR7, KCNH2, MCHR1, NPY1R, OPRD1, OPRK1, OPRL1, OPRM1, PLD1, PLD2, RAMP1, SCARB1, SIGMAR1, SLC22A2,SLC47A1, SLC6A2, SLC6A4, TACR2, TNKS, USP1)

• SwissTargetPrediction tool predicted several adrenergic receptors subtypes (A1A, A1B, A1D, A2A, A2B, A2C) as domperidone targets. The results also retrieved domperidone on-label targets dopamine receptors (D2, D3, D4) as possible targets which increases reliability of these results. Fifteen targets all with high probability scores are predicted by this tool:

(ADRA2A, DRD2, ADRA2B, ADRA2C, DRD4, SLC6A2, ADRA1D, HTR2A, HTR2C, SLC6A4, ADRA1A, HRH1, ADRA1B, OPRM1, DRD3)

• Polypharmacology browser was fed by the SMILES code and the number of targets option was set to its maximum value of 50 targets. The job retrieved 43 targets domperidone already has wet lab determined affinities as curated by ChEMBL. Five targets were filtered as they are cell-lines and organisms. One hit of the results has 2 targets HTR3A_HTR3B.The final wet lab list contained 39 targets:

(ADRA1A, ADRA1B, ADRA1D, ADRA2A, ADRA2B, ADRA2C, ALOX15B, AOX1, ATXN2, CYP2D6, DRD1, DRD2, DRD3, EHMT2, GMNN, HIF1A, HRH1, HTR2A, HTR2B, HTR2C, HTR3A, HTR3B, HTR4, IMPA1, KCNH2, LMNA, NFKB1, OPRK1, OPRM1, PFK, RGS4, SLC22A2, SLC47A1, SLC6A2, SLC6A4, SLCO1B1, SLCO1B3, TXNRD1, USP2).

The insilico predicted targets were 37. Some of them contain more than one target subtype. After filtering targets with significance level (P-value ≤0.05) by any fingerprint, the shortlist contained 21 targets:

(ADORA1, ADORA2A, ADORA2B, ADORA3, CHRM1, CHRM2, CHRM3, CHRM4, CHRM5, CYP2C9, DRD4, DRD5, HDAC4, HDAC6, HTR1A, HTR7, PIN1, PLD1, PLD2, PTGS1, PTGS2).

### Pharmacophore mapping

Domperidone SDF file was fed to the PharMapper server. The job id was (180,718,102,736) and retrieved 232 targets. Targets were sorted by Z-scores and filtered according to significance level of P-value <0.05 (Z-score cutoff ≥1.64). Seventeen targets were selected:

(ABL1, ADH5, AURKA, BACE1, CDK2, CSNK1G2, CTSK, CTSS, EGFR, F2, FNTA, LCK, MAPK14, NOS3, PARP1, PDPK1, REN).

To keep this example brief, molecular docking was done only on the targets filtered by the next step. The final OLT list contained 96 genes that will be used to retrieve the DA list. Visualization of this list by STRING database is available through the link https://version-11-0.string-db.org/cgi/network.pl?networkId=icgrvT0WSen6.

### Retrieving related diseases and ADRs (DA list)

#### Disease enrichment

The OLT list was used in the CTD enrichment analysis tool. The highest enriched disease categories were roughly nervous, cardiovascular and mental disorders.

#### Genes and variants disease associations

The OLT list was fed to DisGeNET as a multiple gene list to retrieve domperidone DA list. A total of 13,493 gene-disease associations and 4,600 variant-disease associations were retrieved. The highest GDA score was 1 for LMNA gene associations to progeria and cardiomyopathy. The highest VDA score was 0.83 for the association of gene KCNH2 reference SNP (rs199472936) to Long Qt Syndrome 2.

### Filtering DA list to get answers

Filtering DA gene list by the ADR name retrieved the list of candidate genes that are related to this ADR. Table 1, Table 2, Table 3, Table 4 show the results of filtration by the ADRs names (arrhythmia, arrest, Torsades de Pointes, and long QT).

**Table 1:** Predicted genes for arrhythmias caused by domperidone.

	Gene	Disease	GDA score
1	KCNH2	Cardiac Arrhythmia	0.4
2	HTR4	Cardiac Arrhythmia	0.3
3	OPRK1	Cardiac Arrhythmia	0.3
4	OPRL1	Cardiac Arrhythmia	0.3
5	PTGS2	Cardiac Arrhythmia	0.3
6	DRD2	Tachyarrhythmia	0.3
7	HTR4	Tachyarrhythmia	0.3
8	LMNA	Ventricular arrhythmia	0.17
9	LMNA	Atrial arrhythmia	0.1
10	LMNA	Cardiac Arrhythmia	0.1
11	GRIN2B	Hypsarrhythmia	0.1
12	LMNA	Primary atrial arrhythmia	0.1
13	LMNA	Supraventricular arrhythmia	0.1
14	KCNH2	Ventricular arrhythmia	0.04
15	CHRM3	Ventricular arrhythmia	0.01
16	CYP2D6	Ventricular arrhythmia	0.01

**Table 2:** Predicted genes for cardiac arrest and death caused by domperidone.

	Gene	Disease	GDA score
1	LMNA	Sudden Cardiac Death	0.4
2	LMNA	Sudden Cardiac Arrest	0.3
3	LMNA	Sudden Cardiac Arrest	0.3
4	KCNH2	Sudden Cardiac Death	0.13
5	KCNH2	Cardiac Arrest	0.12
6	ATXN2	Cardiac Arrest	0.1
7	NFKB1	Cardiac Arrest	0.02
8	EGFR	Cardiac Arrest	0.01
9	LMNA	Cardiac Arrest	0.01
10	PTGS2	Cardiac Arrest	0.01
11	CACNA1G	Cardiac Arrest	0.01
12	HDAC4	Cardiac Arrest	0.01
13	GMNN	Cardiac Arrest	0.01
14	ADRA2B	Sudden Cardiac Death	0.01

**Table 3:** Predicted genes for Torsades de Pointes caused by domperidone.

	Gene	Disease	GDA score
1	KCNH2	Torsades de Pointes	0.5
2	ADRA2C	Torsades de Pointes	0.01
3	CYP2D6	Torsades de Pointes	0.01
4	SLCO1B1	Torsades de Pointes	0.01

**Table 4:** Predicted genes for long QT interval caused by domperidone.

	Gene	Disease	GDA score
1	KCNH2	Acquired long QT syndrome	0.7
2	KCNH2	Congenital long QT syndrome	0.7
3	KCNH2	Long QT Syndrome	0.4
4	KCNH2	LONG QT SYNDROME 1/2, DIGENIC (disorder)	0.3
5	KCNH2	Long Qt Syndrome 2	0.3
6	ADRA1A	Long Qt Syndrome 2	0.3
7	KCNH2	LONG QT SYNDROME 2, ACQUIRED, SUSCEPTIBILITY TO	0.3
8	KCNH2	LONG QT SYNDROME 2/3, DIGENIC	0.2
9	KCNH2	LONG QT SYNDROME 2/5, DIGENIC (disorder)	0.1
10	KCNH2	LONG QT SYNDROME 2/9, DIGENIC	0.1
11	KCNH2	LONG QT SYNDROME 3	0.09
12	KCNH2	Long QT syndrome type 3	0.04
13	KCNH2	LONG QT SYNDROME, BRADYCARDIA-INDUCED	0.01
14	KCNH2	Prolonged QT interval	0.01

It's obvious that KCNH2 is strongly related to all cardiac side effects that are previously reported as side effects of domperidone. This result harmonizes with the previous works of [38,39]. As these cardiovascular disorders are polygenic [41] and may be a result of multiple genes, all associated gene were kept even those with small GDA scores. Deeper analysis may reveal better results to avoid redundancy and disease name differences between different databases.

### Filtering at the genetic variants level

Answers on the variant level could also be retrieved from the DA list. Table 5 shows 27 genetic variants retrieved from domperidone DA list and related to arrhythmias and Torsades de Pointes.

**Table 5:** Predicted genetic variants for arrhythmias and Torsades de Pointes caused by domperidone.

	Disease	Variant ID	consequence	alleles	variant class	Score VDA
1.	Arrhythmogenic Right Ventricular Dysplasia, Familial, 9	rs1114167345	missense variant	A/G	SNP	0.7
2.	Arrhythmogenic Right Ventricular Dysplasia, Familial, 9	rs727505038	missense variant	G/C	SNP	0.7
3.	Cardiac Arrhythmia	rs794728425	frameshift variant	C/CGGGGCGATGGGAGCTGGCCG	in-del	0.7
4.	Cardiac Arrhythmia	rs794728426	frameshift variant	GCGCG/GGCTTTT	in-del	0.7
5.	Cardiac Arrhythmia	rs794728428	frameshift variant	TCGTCGGC/T	in-del	0.7
6.	Cardiac Arrhythmia	rs794728434	frameshift variant	T/TGCAG	in-del	0.7
7.	Cardiac Arrhythmia	rs794728456	frameshift variant	CG/C	in-del	0.7
8.	Cardiac Arrhythmia	rs794728457	frameshift variant	GCTCTCCC/G	in-del	0.7
9.	Cardiac Arrhythmia	rs794728463	frameshift variant	A/AGG	in-del	0.7
10.	Cardiac Arrhythmia	rs794728464	frameshift variant	C/CGCCT	in-del	0.7
11.	Cardiac Arrhythmia	rs794728465	frameshift variant	A/AG	in-del	0.7
12.	Cardiac Arrhythmia	rs794728467	frameshift variant	G/GCCGCC,GCCGC	in-del	0.7
13.	Cardiac Arrhythmia	rs794728469	frameshift variant	G/GCCCC,GCCC,GCC,GC	in-del	0.7
14.	Cardiac Arrhythmia	rs794728470	frameshift variant	A/ACGTCGCCC,ACGTCGC	in-del	0.7
15.	Cardiac Arrhythmia	rs794728472	frameshift variant	TG/T	in-del	0.7
16.	Cardiac Arrhythmia	rs794728476	inframe insertion	C/CCTGCGCGAT	in-del	0.7
17.	Cardiac Arrhythmia	rs794728489	frameshift variant	A/ACCAC	in-del	0.7
18.	Cardiac Arrhythmia	rs794728497	frameshift variant	GC/G	in-del	0.7
19.	Cardiac Arrhythmia	rs794728499	frameshift variant	AG/A	in-del	0.7
20.	Cardiac Arrhythmia	rs794728500	frameshift variant	CG/C	in-del	0.7
21.	Cardiac Arrhythmia	rs794728506	frameshift variant	GC/G	in-del	0.7
22.	Cardiac Arrhythmia	rs794728507	frameshift variant	AC/A	in-del	0.7
23.	Cardiac Arrhythmia	rs794728508	frameshift variant	CA/C	in-del	0.7
24.	Torsades de Pointes	rs1805123	missense variant	T/A,C,G	SNP	0.01
25.	Torsades de Pointes	rs189014161	stop gained	G/A,C,T	SNP	0.01
26.	Torsades de Pointes	rs201268831	stop gained	C/A,T	SNP	0.01
27.	Ventricular arrhythmia	rs56984562	missense variant	C/A,G,T	SNP	0.01

These variants could be used in designing clinical trails that explore domperidone cardiotoxic effects or other similar drug-centered precision medicine research activities.

### Docking

Twenty genes from Table 1, Table 2, Table 3, Table 4 are shortlisted as candidates for domperidone related ADRs (arrhythmias, cardiac arrest, long QT interval, and Torsades de Pointes). Seven targets of these twenty are insilico predicted and the remaining 13 are reported in ChEMBL. Docking jobs of domperidone to these 7 targets (CACNA1G, CHRM3, GRIN2B, HDAC4, OPRL1, PTGS2, EGFR**)** were done using PyRX (version 0.8). Affinities in Kcal/mol were fed to the K_d_ calculator to estimate K_d_s. Resulting affinities were compared to domperidone / DRD3 affinity that was retrieved from Binding DB as calculated by Freedman et al. [42]. The results are represented in Table 6.

**Table 6:** Predicted affinity in nM for selected domperidone predicted targets.

	Symbol	PDB/ SWISS-MODEL ID	Affinity Kcal/mol	K_d_ nM	Domperidone / DRD3 affinity nM
1.	CACNA1G	O43497 (SM)	−5.7	64072	0.58
2.	CHRM3	4DAJ (PDB)	−9.7	73.11	
3.	GRIN2B	4PE5 (PDB)	−5.7	64072	
4.	HDAC4	4CBY (PDB)	−8.6	471.22	
5.	OPRL1	5DHG (PDB)	−8.5	558.2	
6.	PTGS2	5F1A (PDB)	−10.1	37.13	
7.	EGFR	3BEL (PDB)	−7.7	2164.34	

Docking results could be used for further filtering of the previous results to select targets that are more likely involved in certain ADRs. For example, Table 6 results could suggest the selection of CHRM3 and PTGS2 for more wet lab analysis to explore their relation to the previously mentioned domperidone cardiotoxic ADRs.

### Example 2: ranitidine related thrombocytopenia

Ranitidine is a well-known H2 receptor antagonist drug that is commonly used for treatment of gastric acid related diseases. Ranitidine is listed under drug bank accession No. (DB00863). One of the most reported ranitidine ADRs in VigiAccess is thrombocytopenia. Feeding PHARMIP with ranitidine SDF file and SMILES code from the DrugBank in exploration of this ADR underpinning genetics retrieved the following results:

### Predicting the off-label drug targets (OLT list)

SEA server

• ACHE, BCHE, HRH2, QPCTL, QPCT, SLC47A1

SwissTargetPrediction

• BCHE, ACHE, HRH2, CHRM2, CHRM4, CHRM1

Poly-pharmacology

• Wet (PMP22, ACHE, LMNA, BLM, NFKB1, HIF1A, SLC47A1, ATP4B, ATP4A, ATP12A, TSHR, HRH2, HSD17B10)
• Dry (GMNN, POLI, CHRM1, BCHE, CHRM2, CHRM2, SLC22A1, TYMP, ITGA2B, ITGB3)

PharMapper (Job ID: 191,116,114,142)

• (HRAS, TGM3, PTPN1, RAF1, RAC1, MAPK10, BHMT, PMS2, RAN, AMPM2, RORA, AK1, GSTA1, CDK2, ARHGAP1, ANG, CA2, AKR1B1, OAT, LCK, NNT, SRC, GSTP1, HSP90AA1, PIM1, C8G, SHMT1, GLO1, DTYMK, F2, ADH5, UCK2, SDS, MMP3, DDX39B, GPI, ITPKA, NME2, SSE1, GCK, HSPA1B, PTK2, RAB5A)

Final OLT list

• (ACHE, ADH5, AK1, AKR1B1, AMPM2, ANG, ARHGAP1, ATP12A, ATP4A, ATP4B, BCHE, BHMT, BLM, C8G, CA2, CDK2, CHRM1, CHRM2, CHRM4, DDX39B, DTYMK, F2, GCK, GLO1, GMNN, GPI, GSTA1, GSTP1, HIF1A, HRAS, HRH2, HSD17B10, HSP90AA1, HSPA1B, ITGA2B, ITGB3, ITPKA, LCK, LMNA, MAPK10, MMP3, NFKB1, NME2, NNT, OAT, PIM1, PMP22, PMS2, POLI, PTK2, PTPN1, QPCT, QPCTL, RAB5A, RAC1, RAF1, RAN, RORA, SDS, SHMT1, SLC22A1, SLC47A1, SRC, SSE1, TGM3, TSHR, TYMP, UCK2)

The OLT list could be visualized using the link https://version-11-0.string-db.org/cgi/network.pl?networkId=CsijWpejaTh1.

### Retrieving related diseases and ADRs (DA list)

The enrichment analysis using OLT gene list retrieved number of related diseases like cancer, digestive system diseases, and cardiovascular diseases. The related diseases list retrieved by DisGeNet contained 8959 entry and 3423 entry for the variant's diseases list.

### Filtering DA list to get answers

Filtering the genes and variants lists that are related to ranitidine OLT list and thrombocytopenia retrieved the following tables (7, 8 and 9):

**Table 7:** Predicted genes for thrombocytopenia caused by ranitidine.

	Gene	Disease	GDA score
1.	SRC	THROMBOCYTOPENIA 6	0.7
2.	ITGB3	Neonatal Alloimmune Thrombocytopenia	0.56
3.	ITGB3	Thrombocytopenia	0.37
4.	ITGA2B	Thrombocytopenia	0.34
5.	ITGA2B	Neonatal Alloimmune Thrombocytopenia	0.31
6.	ITGB3	Autosomal dominant macrothrombocytopenia	0.31
7.	ITGA2B	Autosomal dominant macrothrombocytopenia	0.3
8.	ITGA2B	Other primary thrombocytopenia	0.2
9.	ITGB3	Other primary thrombocytopenia	0.2
10.	ITGB3	Macrothrombocytopenia	0.14
11.	ITGA2B	Macrothrombocytopenia	0.11
12.	SRC	Thrombocytopenia	0.11
13.	CA2	Thrombocytopenia	0.1
14.	NFKB1	Autoimmune thrombocytopenia	0.1
15.	NFKB1	Idiopathic thrombocytopenia	0.1
16.	ACHE	Thrombocytopenia	0.02
17.	BCHE	Thrombocytopenia	0.02
18.	ITGA2B	Neonatal thrombocytopenia (disorder)	0.01
19.	ITGA2B	Congenital amegakaryocytic thrombocytopenia	0.01
20.	ITGB3	Autoimmune thrombocytopenia	0.01
21.	ITGB3	THROMBOCYTOPENIA 2 (disorder)	0.01
22.	SLC22A1	Thrombocytopenia	0.01

**Table 8:** Predicted variants for ranitidine related thrombocytopenia.

	Disease	Variant ID	Gene	Consequence	Alleles	Score VDA
1.	THROMBOCYTOPENIA 6	rs879255268	SRC	missense variant	G/A	0.8
2.	Neonatal Alloimmune Thrombocytopenia	rs547581737	ITGA2B	missense variant	G/A	0.01
3.	Neonatal thrombocytopenia (disorder)	rs547581737	ITGA2B	missense variant	G/A	0.01
4.	Macrothrombocytopenia	rs761164933	ITGA2B	missense variant	G/A	0.01

**Table 9:** Predicted affinities in nM for selected ranitidine predicted targets.

	Symbol	PDB/ SWISS-MODEL ID	Affinity Kcal/mol	K_d_ nM	Ranitidine/ H2 affinity nM
1.	BCHE	1EHO (PDB)	−10.9	9.58	64.4
2.	CA2	2QP6 (PDB)	−9.2	170.54	
3.	ITGA2B	5THP (PDB)	−8.8	335.8	
4.	ITGB3	1JV2 (PDB)	−7.7	2164.34	
5.	SLC22A1	O15245 (SM)	−7.9	1542.38	
6.	SRC	1A09 (PDB)	−9.2	170.54	

On the genetic variant level, the next table contains the variants of interest that can be used in drug-centered precision medicine research activities:

### Docking

A list of 8 genes (ACHE, BCHE, CA2, ITGA2B, ITGB3, NFKB1, SLC22A1, SRC) was predicted to underpin the ranitidine related thrombocytopenia. Two of them (ACHE, NFKB1) were already reported in ChEMBL as ranitidine targets through wet lab experiments. The results of ranitidine docking to the other 6 targets is summarized in the following table: based on this rough estimation, (BCHE, CA2, SRC) could be suitable candidates for further ranitidine related thrombocytopenia wet lab invistigations.

## Supplementary material *and/or* Additional information

The following supplementary materials are attached to the paper:

• Two Excel workbooks that contain all the results retrieved by the insilico tools and dependent analysis results.
• Two Word documents contain the refined gene lists retrieved by the insilico tools and formatted as inputs for DisGeNET and CTD analyzer.
• Two Zip folders contain the files used for docking and the python script to calculate K_d_s.

## Declaration of Competing Interest

The authors declare no conflicts of interest in preparing this manuscript.
