# Supplementary material for: PHARMIP: An insilico method to predict genetics that underpin adverse drug reactions
Source: MethodsX. 2019 Dec 19;7:100775. doi: 10.1016/j.mex.2019.100775 (PMC7036477; doi:10.1016/j.mex.2019.100775)
Supplement: Supplementary file 1 [file mmc1.zip › results Ranitidine.docx]

List of predicted targets for Ranitidine

SEA (SMILES code : CNC(NCCSCC1=CC=C(CN(C)C)O1)=C[N+]([O-])=O)

- ACHE, BCHE, HRH2, QPCTL, QPCT, SLC47A1

SwissTargetPrediction

- BCHE , ACHE ,HRH2, CHRM2, CHRM4, CHRM1

Poly-pharmacology

- Wet (PMP22 , ACHE ,LMNA ,BLM ,NFKB1 ,HIF1A ,SLC47A1 ,ATP4B, ATP4A, ATP12A ,TSHR ,HRH2 , HSD17B10)
- Dry (GMNN, POLI , CHRM1 ,BCHE ,CHRM2 ,CHRM2 , SLC22A1 ,TYMP ,ITGA2B, ITGB3)

PharMapper (Job ID : 191116114142)

- ( HRAS, TGM3, PTPN1, RAF1, RAC1, MAPK10, BHMT, PMS2, RAN, AMPM2, RORA, AK1, GSTA1, CDK2, ARHGAP1, ANG, CA2, AKR1B1, OAT, LCK, NNT, SRC, GSTP1, HSP90AA1, PIM1, C8G, SHMT1, GLO1, DTYMK, F2, ADH5, UCK2, SDS, MMP3, DDX39B, GPI, ITPKA, NME2, SSE1, GCK, HSPA1B, PTK2, RAB5A)

Final OLT list

- ( ACHE:: ADH5:: AK1:: AKR1B1:: AMPM2:: ANG:: ARHGAP1:: ATP12A:: ATP4A:: ATP4B:: BCHE:: BHMT:: BLM:: C8G:: CA2:: CDK2:: CHRM1:: CHRM2:: CHRM4:: DDX39B:: DTYMK:: F2:: GCK:: GLO1:: GMNN:: GPI:: GSTA1:: GSTP1:: HIF1A:: HRAS:: HRH2:: HSD17B10:: HSP90AA1:: HSPA1B:: ITGA2B:: ITGB3:: ITPKA:: LCK:: LMNA:: MAPK10:: MMP3:: NFKB1:: NME2:: NNT:: OAT:: PIM1:: PMP22:: PMS2:: POLI:: PTK2:: PTPN1:: QPCT:: QPCTL:: RAB5A:: RAC1:: RAF1:: RAN:: RORA:: SDS:: SHMT1:: SLC22A1:: SLC47A1:: SRC:: SSE1:: TGM3:: TSHR:: TYMP:: UCK2)
